# *Arabidopsis thaliana* protein NSL1 interacts with *Pseudomonas syringae* pv. *tomato* DC3000 effector HopM1 in a yeast 2-hybrid assay

**DOI:** 10.17912/micropub.biology.001311

**Published:** 2024-09-13

**Authors:** Irina Sementchoukova, Ana Domínguez-Ferreras, Vardis Ntoukakis, Jacqueline Monaghan

**Affiliations:** 1 Department of Biology, Queen's University, Kingston, Ontario, Canada; 2 School of Life Sciences, University of Warwick, Coventry, England, United Kingdom

## Abstract

*Arabidopsis thaliana* proteins NECROTIC SPOTTED LESIONS 1 ( NSL1 ) and CONSTITUTIVE ACTIVE DEFENSE 1 ( CAD1 ) have previously been linked to immunity against phytopathogens such as *Pseudomonas syringae* pv. *tomato* ( *Pst* ) DC3000 (Noutoshi et al. 2006; Tsutsui et al. 2008; Asada et al. 2011; Fukunaga et al. 2017; Holmes et al. 2021). Here, we used a yeast 2-hybrid (Y2H) approach to explore their potential to interact with *Pst* DC3000 effectors. We found that NSL1 , but not CAD1 , interacted with the *Pst* DC3000 effector HopM1. Although further experiments are needed to validate this interaction, our results suggest that NSL1 may be a host target of HopM1.

**Figure 1. Y2H screen identifies Arabidopsis protein NSL1 as an interactor of Pst DC3000 effector HopM1:**
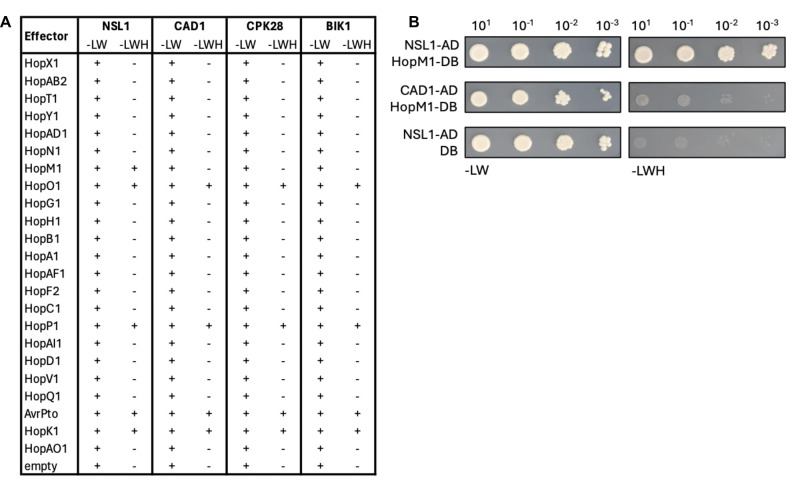
**(A)** Table summarizing the results of a pairwise yeast 2-hybrid interaction screen between 23 *Pst* DC3000 effectors and Arabidopsis proteins NSL1 , CAD1 , CPK28 , and BIK1 . Growth (+) of AH109 yeast cells on media lacking Leu and Trp (-LW) indicates successful co-transformation of bait and prey plasmids, while growth (+) on media lacking Leu, Trp, and His (-LWH) indicates activation of the His biosynthesis marker gene. No growth (-) on -LWH media indicates no activation of the reporter gene. The screen was completed twice with the same results. **(B)** Dilution series of AH109 cells independently co-transformed with NSL1-AD and HopM1-DB indicates strong growth on both -LW and -LWH media, while AH109 cells independently co-transformed with CAD1-AD and HopM1-DB, or NSL1-AD and empty DB vector only grow on -LW media. This experiment was repeated three times with the same results.

## Description

Arabidopsis proteins NSL1 (At1g28380) and CAD1 ( At1g29690 ) contain domains with homology to membrane attack complex (MAC) and perforin (PF) proteins (Noutoshi et al. 2006; Tsutsui et al. 2008; Fukunaga et al. 2017; Holmes et al. 2021) . In mammals, MACPF proteins form pores in plasma membranes and are well-known and critical components of adaptive immunity (Lukoyanova et al. 2016) . While the ability of MACPF proteins to form pores in plant cell membranes has not been shown, both NSL1 and CAD1 are likely to be involved in the plant immune response since loss-of-function *nsl1* and *cad1* mutants display hallmarks of autoimmunity such as high levels of salicylate, enhanced expression of pathogenesis-related genes, and enhanced resistance against the bacterial pathogen *Pseudomonas syringae* pv. *tomato* ( *Pst* ) DC3000 (Noutoshi et al. 2006; Tsutsui et al. 2008; Fukunaga et al. 2017; Holmes et al. 2021) . Pathogens such as *Pst* DC3000 secrete effector proteins into host cells to interfere with immune signaling (Wang et al. 2022) . The presence of pathogen effectors is detected by cytoplasmic nucleotide binding leucine rich repeat (NLR) receptors that re-localize to the plasma membrane as pore-forming resistosomes resulting in a form of programmed cell death known as the hypersensitive response (HR) (Huang et al. 2023) . Because *nsl1* and *cad1* mutants display constitutive cell death reminiscent of uncontrolled HR, which can be suppressed by genetically blocking NLR signaling, we and others hypothesized that NSL1 and CAD1 may be targeted by pathogen effectors and that their integrity may be guarded by NLRs (Noutoshi et al. 2006; Tsutsui et al. 2008; Fukunaga et al. 2017; Holmes et al. 2021) .

Previous studies have used the yeast 2-hybrid (Y2H) approach to map the Arabidopsis interactome of effectors from unrelated pathogen species (Mukhtar et al. 2011; Weßling et al. 2014) . A main conclusion from these studies was that effector proteins from diverse pathogen kingdoms tend to interact with an overlapping range of host targets. To test the hypothesis that NSL1 and/or CAD1 may be directly targeted by pathogen effectors, we conducted a Y2H screen to test for association between NSL1 , CAD1 , and 23 effectors from the bacterial phytopathogen *Pseudomonas syringae* pv. *tomato* ( *Pst* ) DC3000. The Y2H method is based on the principle that the yeast Gal4 transcription factor can be split into its DNA binding (BD) and DNA activation (AD) domains (Fields and Song 1989) . When 'bait' and 'prey' proteins are translationally fused to the BD and AD domains and expressed in yeast, interaction between the proteins reconstitutes Gal4 and drives the expression of reporter genes. The yeast strain AH109 is auxotrophic for the ability to synthesize amino acids Leu, Trp, and His. The ‘bait' and ‘prey' plasmid vectors carry the genetic capacity to confer Leu and Trp biosynthesis, respectively, and reconstituted Gal4 drives transcription of the His biosynthesis gene, thus allowing for the selection of interacting protein binding partners via growth on media lacking Leu, Trp, and His. Here, we cloned 23 *Pst* DC3000 effectors as baits in frame with Gal4-DB, and cloned NSL1 and CAD1 as preys in frame with the Gal4-AD. NSL1 and CAD1 were transformed into AH109 with each of the 23 *Pst* DC3000 effectors in a pairwise manner. Successful transformants were obtained for NSL1 , CAD1 , and each of the *Pst* DC3000 effector pairs, as is demonstrated by growth on media lacking Leu and Trp ( **Figure 1A** ). We found that both NSL1 and CAD1 co-transformed with effectors HopO1, HopP1, AvrPto, and HopK1 exhibited strong growth when re-plated on restrictive media lacking His ( **Figure 1A** ), suggesting potential positive interactions. However, HopO1, HopP1, AvrPto, and HopK1 also exhibited strong growth on restrictive media when co-transformed with two other unrelated Arabidopsis prey proteins that we employed as controls - BOTRYTIS INDUCED KINASE 1 ( BIK1 ; At2g39660) and CALCIUM DEPENDENT PROTEIN KINASE 28 ( CPK28 ; At5g66210) ( **Figure 1A** ). The promiscuous range of interactions displayed by these baits suggests that they are false-positives. We therefore discounted NSL1 and CAD1 as putative interactors of HopO1, HopP1, AvrPto, and HopK1. Conversely, we found that yeast co-transformed with HopM1 and NSL1 were able to grow on media lacking His, which was not observed when HopM1 was co-transformed with CAD1 , BIK1 or CPK28 ( **Figure 1A** ). We further confirmed this interaction in independent transformations alongside controls ( **Figure 1B** ). Together, our results indicate that HopM1 and NSL1 can associate with each other in the Y2H system, providing preliminary evidence that NSL1 may be a novel target of the *Pst* DC3000 effector HopM1.

HopM1 is a 712-amino acid protein belonging to the minimal effector repertoire of *Pst* DC3000 required to promote pathogenicity (Cunnac et al. 2011) that suppresses disease resistance in *Nicotiana benthamiana* (Oh and Collmer 2005) and Arabidopsis (Nomura et al. 2006, 2011; Lozano-Durán et al. 2014). Furthermore, HopM1 induces leaf-soaking and proteophagy in Arabidopsis (Xin et al. 2016; Üstün et al. 2018; Roussin-Léveillée et al. 2022). In a previous Y2H screen using a truncated variant of HopM1 containing only its N-terminus (HopM1 ^1-300^ ), 21 unrelated proteins from Arabidopsis were identified and named as Arabidopsis HopM1-interactors (AtMINs). Interestingly, co-expression of selected AtMIN proteins with full-length HopM1 ^1-712^ resulted in their degradation in yeast cells, suggesting that HopM1 interacts with AtMINs via its N terminus and mediates their degradation via its C terminus (Nomura et al. 2006) . We detected interaction between HopM1 ^1-712^ and NSL1 , which suggests that NSL1 is not degraded in yeast and points towards a different mechanism.

One target of HopM1 is the endomembrane-localized adenosine diphosphate ribosylation factor guanine nucleotide exchange factor protein AtMIN7 (At3g43300), allowing it to intercept protein trafficking (Nomura et al. 2006, 2011) . Notably, AtMIN7 is genetically required for the immune-induced accumulation of CAD1 , and AtMIN7 and CAD1 were recently shown to contribute to microbial community composition in the phyllosphere (Chen et al. 2020) . NSL1 similarly localizes to the plasma membrane and accumulates following detection of the immunogenic peptide flg22 (Fukunaga et al. 2017) . As part of other work, we identified putative NSL1 binding partners following affinity purification of NSL1-YFP from *nsl1-1/35S:NSL1-YFP* transgenic lines compared to controls (Dias et al. 2023) . Although preliminary, we identified AtMIN7 as a putative association partner of NSL1 (Dias et al. 2023) . While CAD1 did not interact with HopM1 in the Y2H assay, the potential association of AtMIN7 with both CAD1 (Chen et al. 2020) and NSL1 (Dias et al. 2023) suggests a possible connection between HopM1 target proteins.

Overall, we identified the Arabidopsis protein NSL1 as a putative novel interactor of *Pst* DC3000 effector HopM1 in a Y2H experiment. Further work is necessary to determine if this interaction occurs *in planta* during an infection, and if so, how this interaction contributes to pathogen virulence or host immunity.

## Methods

**Molecular cloning:** The open reading frames of *CAD1* , *NSL1* , *CPK28* , and *BIK1* were PCR-amplified from existing plasmids using Q5 Taq Polymerase (New England Biolabs; NEB), and cloned into Gateway-compatible pENTR entry vectors using Gibson Assembly Master Mix (NEB) according to the manufacturer's instructions (Gibson et al. 2009) . The Gateway-compatible *Pst* DC3000 effector library in pEarlyGate201 was previously described (Gimenez-Ibanez et al. 2018) . Individual effectors were shuttled from pEarlyGate201 into pDONR207 using BP Clonase (Invitrogen) according to the manufacturer's instructions. LR Clonase II Enzyme Mix (Invitrogen) was used to shuttle inserts from the pENTR or pDONR207 entry vectors into the pDEST22 (Invitrogen, ProQuestTM Two Hybrid System) or the pDEST-DB destination vectors (Dreze et al. 2010) according to the manufacturer's instructions. All vectors were verified by Sanger sequencing (The Genome Analysis Centre, Toronto; Eurofins Genomics Europe, Germany). All vectors, primers, and reagents used in this study are described in **Tables 1-3** .

**Yeast 2-hybrid:** Auxotrophic AH109 yeast cells were co-transformed with Gal4-DNA Binding Domain (pDEST-DB) and Gal4-AD (pDEST22) plasmids carrying *Pst* DC3000 effectors and Arabidopsis proteins respectively. AH109 cells were cultured in liquid yeast peptone dextrose media (YPD; Bioshop) and grown until mid-logarithmic phase (OD _546_ 0.6 – 1.0) at 30⁰C. Cells were pelleted by centrifugation at 1,592 x *g* for 5 minutes, washed with sterile water, resuspended in 100 mM LiAc, and incubated at 30⁰C for 10 minutes. After incubation, cells were aliquoted into individual tubes and mixed in a 1:3 ratio with transformation buffer (33% (v/v) PEG-3350; 100 mM LiAc; 0.27 mg/mL boiled single stranded salmon sperm DNA (Sigma-Aldrich); 10% (v/v) DMSO), along with 100 ng of each of the pDEST-DB and pDEST22 plasmids. The cells were lightly vortexed to mix, incubated at 30⁰C for 30 minutes, and then transferred to 42⁰C for 40 minutes. Transformed cells were pelleted by gentle centrifugation for 1 minute and resuspended in water, plated onto pre-warmed agar plates lacking Leu and Trp (-LW) containing yeast nitrogen base without amino acids (Bioshop), yeast synthetic drop-out medium supplements without His, Leu, Trp, and Ade (Sigma Aldrich), 2% (v/v) glucose, 0.8 mM histidine-HCl, and 150 mg/L adenine sulfate (Bioshop). Selection plates lacking His (-LWH) were made without the addition of Histidine-HCl. After three days of recovery on -LW plates, individual yeast colonies were subcultured on fresh -LW and -LWH plates, incubated at 30⁰C, and grown for three days. Cells grown on -LW plates were used to inoculate liquid -LW media, cultured overnight at 30⁰C, diluted to an OD _546_ of 0.1, followed by serially 10-fold diluted. 10 µL of each dilution was dropped onto -LW and -LWH plates using a multi-channel pipette and incubated at 30⁰C for three days. All reagents used in this study are described in **Table 3** .

&nbsp;

&nbsp;

&nbsp;

&nbsp;

&nbsp;

&nbsp;

&nbsp;

## Reagents

**Table 1: Clones used in this study.**

**Table:** 

**Name**	**Gene ID**	**Notes**	**Reference**
pDEST22- NSL1	At1g28380	Gal4AD-tagged NSL1 ( *A. thaliana* )	This study; cloned by IS.
pENTR- NSL1 (NP/NS)	At1g28380	pENTR entry clone containing the open reading frame of NSL1 ( *A. thaliana* ) with no promoter (NP) and no stop codon (NS)	(Holmes et al. 2021)
pDEST22- CAD1	At1g29690	Gal4AD-tagged CAD1 ( *A. thaliana* )	This study; cloned by IS.
pENTR- CAD1 (NP/OS)	At1g29690	pENTR entry clone containing the open reading frame of CAD1 ( *A. thaliana* ) with no promoter (NP) and endogenous stop codon (OS)	This study; cloned by IS.
pDEST22- BIK1	At2g39660	Gal4AD-tagged BIK1 ( *A. thaliana* )	This study; cloned by IS.
pENTR- BIK1 (NP/NS)	At2g39660	pENTR entry clone containing the open reading frame of BIK1 ( *A. thaliana* ) with no promoter (NP) and no stop codon (NS)	This study; cloned by IS.
pDEST22- CPK28	At5g66210	Gal4AD-tagged CPK28 ( *A. thaliana* )	This study; cloned by IS.
pENTR- CPK28 (NP/NS)	At5g66210	pENTR entry clone containing the open reading frame of CPK28 ( *A. thaliana* ) with no promoter (NP) and no stop codon (NS)	(Monaghan et al. 2014)
pDONR207 and pDEST-DB-HopO1	PSPTO_A0018	Gal4BD-tagged HopO1 ( *Pst* DC3000)	This study; cloned by ADF.
pDONR207 and pDEST-DB-HopM1	PSPTO_1375	Gal4BD-tagged HopM1 ( *Pst* DC3000)	This study; cloned by ADF.
pDONR207 andpDEST-DB-HopN1	PSPTO_1370	Gal4BD-tagged HopN1 ( *Pst* DC3000)	This study; cloned by ADF.
pDONR207 and pDEST-DB-HopAD	PSPTO_4691	Gal4BD-tagged HopAD ( *Pst* DC3000)	This study; cloned by ADF.
pDONR207 and pDEST-DB-HopY	PSPTO_1372	Gal4BD-tagged HopY ( *Pst* DC3000)	This study; cloned by ADF.
pDONR207 and pDEST-DB-HopT	PSPTO_A0019	Gal4BD-tagged HopT ( *Pst* DC3000)	This study; cloned by ADF.
pDONR207 and pDEST-DB-HopAB2	PSPTO_3087	Gal4BD-tagged HopAB2 ( *Pst* DC3000)	This study; cloned by ADF.
pDONR207 and pDEST-DB-HopX	PSPTO_A0012	Gal4BD-tagged HopX ( *Pst* DC3000)	This study; cloned by ADF.
pDONR207 and pDEST-DB-HopP1	PSPTO_2678	Gal4BD-tagged HopP1 ( *Pst* DC3000)	This study; cloned by ADF.
pDONR207 and pDEST-DB-HopC1	PSPTO_0589	Gal4BD-tagged HopC1 ( *Pst* DC3000)	This study; cloned by ADF.
pDONR207 and pDEST-DB-HopF2	PSPTO_0502	Gal4BD-tagged HopF2 ( *Pst* DC3000)	This study; cloned by ADF.
pDONR207 and pDEST-DB-HopAF1	PSPTO_1568	Gal4BD-tagged HopAF1 ( *Pst* DC3000)	This study; cloned by ADF.
pDONR207 and pDEST-DB-HopA1	PSPTO_5354	Gal4BD-tagged HopA1 ( *Pst* DC3000)	This study; cloned by ADF.
pDONR207 and pDEST-DB-HopB1	PSPTO_1406	Gal4BD-tagged HopB1 ( *Pst* DC3000)	This study; cloned by ADF.
pDONR207 and pDEST-DB-HopH1	PSPTO_0588	Gal4BD-tagged HopH1 ( *Pst* DC3000)	This study; cloned by ADF.
pDONR207 and pDEST-DB-HopG1	PSPTO_4727	Gal4BD-tagged HopG1 ( *Pst* DC3000)	This study; cloned by ADF.
pDONR207 and pDEST-DB-HopAO1	PSPTO_4722	Gal4BD-tagged HopAO1 ( *Pst* DC3000)	This study; cloned by ADF.
pDONR207 and pDEST-DB-HopK1	PSPTO_0044	Gal4BD-tagged HopK1 ( *Pst* DC3000)	This study; cloned by ADF.
pDONR207 and pDEST-DB-AvrPto	PSPTO_4001	Gal4BD-tagged AvrPto ( *Pst* DC3000)	This study; cloned by ADF.
pDONR207 and pDEST-DB-HopQ1	PSPTO_0877	Gal4BD-tagged HopQ1 ( *Pst* DC3000)	This study; cloned by ADF.
pDONR207 and pDEST-DB-HopV1	PSPTO_4720	Gal4BD-tagged HopV1 ( *Pst* DC3000)	This study; cloned by ADF.
pDONR207 and pDEST-DB-HopD1	PSPTO_0876	Gal4BD-tagged HopD1 ( *Pst* DC3000)	This study; cloned by ADF.
pDONR207 and pDEST-DB-HopAI1	PSPTO_0906	Gal4BD-tagged HopAI1 ( *Pst* DC3000)	This study; cloned by ADF.

**Table 2: Primers used in this study.**

**Table:** 

**Primer name**	**Primer sequence (5'-3')**	**Reference**
JMo126_pENTRGA_RB	AAGGGTGGGCGCGCCGACCCAG	This study; designed by JM.
JMo339_pENTRGA_LB	GGTGAAGGGGGCGGCCGCGG	This study; designed by JM.
JMo676_pENTRGA_ CAD1 _RB	GGGTCGGCGCGCCCACCCTTTCAATAATTTAGCAACGAATACTT	This study; designed by IS.
JMo677_pENTRGA_ CAD1 _LB	CCGCGGCCGCCCCCTTCACCATGGAGAATCGTAAAGGAGG	This study; designed by IS.
JMo576_pENTRGA_c BIK1 _RB	GGGTCGGCGCGCCCACCCTTCTACACAAGGTGCCTGCCAA	This study; designed by IS.
JMo575_pENTRGA_c BIK1 _LB	CCGCGGCCGCCCCCTTCACCATGGGTTCTTGCTTCAGTTC	This study; designed by IS.

**Table 3: Reagents used in this study.**

**Table:** 

**Item name**	**Vendor (catalog number)**
GenepHlow Gel/PCR Kit	GeneAid (DFH300)
Q5 *Taq* Polymerase	New England Biolabs (M0491)
*Dpn* I endonuclease	New England Biolabs (R0176)
Gibson Assembly Master Mix	New England Biolabs (E2611)
Gateway LR Clonase II Enzyme Mix	Invitrogen (11791020)
Gateway BP Clonase II Enzyme Mix	Invitrogen (11789020)
Agar	BioShop Canada (AGR001)
LB Broth (Miller)	BioShop Canada (LBL407)
Carbenicillin disodium salt	Sigma-Aldrich (C1389)
Gentamicin sulfate salt	Sigma-Aldrich (G3632)
Yeast Peptone Dextrose (YPD)	Sigma-Aldrich (Y1375)
Polyethylene glycol (PEG-3350)	Sigma-Aldrich (1546547)
Lithium acetate (LiAc)	Sigma-Aldrich (920320)
Single-stranded Salmon Sperm DNA	Sigma-Aldrich (D7656)
Dimethylsulfoxide (DMSO)	Sigma-Aldrich (D8418)
Yeast Nitrogen Base Without Amino Acids, Without Ammonium Sulfate	BioShop Canada (YNB404)
Yeast Synthetic Drop-Out Medium Supplements without His, Leu, Trp, Ade	Sigma-Aldrich (Y2021)
Adenine sulfate	Sigma-Aldrich (A3159)
Histidine-HCl	Sigma-Aldrich (43011)
D-Glucose	BioShop Canada (GLU501)
Presto Mini Plasmid Kit	Geneaid (PDF100)
ZymoPURE Plasmid Miniprep Kit	Zymo Research (D4210)

## References

[R1] Asada Yutaka, Yamamoto Masako, Tsutsui Tomokazu, Yamaguchi Junji (2011). The Arabidopsis NSL2 negatively controls systemic acquired resistance via hypersensitive response. Plant Biotechnology.

[R2] Chen Tao, Nomura Kinya, Wang Xiaolin, Sohrabi Reza, Xu Jin, Yao Lingya, Paasch Bradley C., Ma Li, Kremer James, Cheng Yuti, Zhang Li, Wang Nian, Wang Ertao, Xin Xiu-Fang, He Sheng Yang (2020). A plant genetic network for preventing dysbiosis in the phyllosphere. Nature.

[R3] Cunnac Sébastien, Chakravarthy Suma, Kvitko Brian H., Russell Alistair B., Martin Gregory B., Collmer Alan (2011). Genetic disassembly and combinatorial reassembly identify a minimal functional repertoire of type III effectors in
*Pseudomonas syringae*. Proceedings of the National Academy of Sciences.

[R4] Dias Márcia Gonçalves, Doss Bassem, Rawat Anamika, Siegel Kristen R., Mahathanthrige Tharika, Sklenar Jan, Derbyshire Paul, Dharmasena Thakshila, Cameron Emma, Zipfel Cyril, Menke Frank L.H., Monaghan Jacqueline (2023). Subfamily C7 Raf-like kinases MRK1, RAF26, and RAF39 regulate immune homeostasis and stomatal opening in
*Arabidopsis thaliana*.

[R5] Dreze Matija, Monachello Dario, Lurin Claire, Cusick Michael E., Hill David E., Vidal Marc, Braun Pascal (2010). High-Quality Binary Interactome Mapping. Methods in Enzymology.

[R6] Fields Stanley, Song Ok-kyu (1989). A novel genetic system to detect protein–protein interactions. Nature.

[R7] Fukunaga Satoshi, Sogame Miho, Hata Masaki, Singkaravanit‐Ogawa Suthitar, Piślewska‐Bednarek Mariola, Onozawa‐Komori Mariko, Nishiuchi Takumi, Hiruma Kei, Saitoh Hiromasa, Terauchi Ryohei, Kitakura Saeko, Inoue Yoshihiro, Bednarek Paweł, Schulze‐Lefert Paul, Takano Yoshitaka (2017). Dysfunction of Arabidopsis MACPF domain protein activates programmed cell death via tryptophan metabolism in MAMP‐triggered immunity. The Plant Journal.

[R8] Gibson Daniel G, Young Lei, Chuang Ray-Yuan, Venter J Craig, Hutchison Clyde A, Smith Hamilton O (2009). Enzymatic assembly of DNA molecules up to several hundred kilobases. Nature Methods.

[R9] Gimenez-Ibanez Selena, Hann Dagmar R., Chang Jeff H., Segonzac Cécile, Boller Thomas, Rathjen John P. (2018). Differential Suppression of Nicotiana benthamiana Innate Immune Responses by Transiently Expressed Pseudomonas syringae Type III Effectors. Frontiers in Plant Science.

[R10] Holmes Danalyn R, Bredow Melissa, Thor Kathrin, Pascetta Sydney A, Sementchoukova Irina, Siegel Kristen R, Zipfel Cyril, Monaghan Jacqueline (2021). A novel allele of the
*Arabidopsis thaliana*
MACPF protein CAD1 results in deregulated immune signaling. Genetics.

[R11] Huang Shijia, Jia Aolin, Ma Shoucai, Sun Yue, Chang Xiaoyu, Han Zhifu, Chai Jijie (2023). NLR signaling in plants: from resistosomes to second messengers. Trends in Biochemical Sciences.

[R12] Kondos S. C., Hatfaludi T., Voskoboinik I., Trapani J. A., Law R. H. P., Whisstock J. C., Dunstone M. A. (2010). The structure and function of mammalian membrane-attack complex/perforin-like proteins. Tissue Antigens.

[R13] Lozano‐Durán Rosa, Bourdais Gildas, He Sheng Yang, Robatzek Silke (2013). The bacterial effector HopM1 suppresses PAMP‐triggered oxidative burst and stomatal immunity. New Phytologist.

[R14] Lukoyanova Natalya, Hoogenboom Bart W., Saibil Helen R. (2016). The membrane attack complex, perforin and cholesterol-dependent cytolysin superfamily of pore-forming proteins. Journal of Cell Science.

[R15] Monaghan Jacqueline, Matschi Susanne, Shorinola Oluwaseyi, Rovenich Hanna, Matei Alexandra, Segonzac Cécile, Malinovsky Frederikke Gro, Rathjen John P., MacLean Dan, Romeis Tina, Zipfel Cyril (2014). The Calcium-Dependent Protein Kinase CPK28 Buffers Plant Immunity and Regulates BIK1 Turnover. Cell Host & Microbe.

[R16] Mukhtar M. Shahid, Carvunis Anne-Ruxandra, Dreze Matija, Epple Petra, Steinbrenner Jens, Moore Jonathan, Tasan Murat, Galli Mary, Hao Tong, Nishimura Marc T., Pevzner Samuel J., Donovan Susan E., Ghamsari Lila, Santhanam Balaji, Romero Viviana, Poulin Matthew M., Gebreab Fana, Gutierrez Bryan J., Tam Stanley, Monachello Dario, Boxem Mike, Harbort Christopher J., McDonald Nathan, Gai Lantian, Chen Huaming, He Yijian, Vandenhaute Jean, Roth Frederick P., Hill David E., Ecker Joseph R., Vidal Marc, Beynon Jim, Braun Pascal, Dangl Jeffery L., European Union Effectoromics Consortium (2011). Independently Evolved Virulence Effectors Converge onto Hubs in a Plant Immune System Network. Science.

[R17] Nomura Kinya, DebRoy Sruti, Lee Yong Hoon, Pumplin Nathan, Jones Jonathan, He Sheng Yang (2006). A Bacterial Virulence Protein Suppresses Host Innate Immunity to Cause Plant Disease. Science.

[R18] Nomura Kinya, Mecey Christy, Lee Young-Nam, Imboden Lori Alice, Chang Jeff H., He Sheng Yang (2011). Effector-triggered immunity blocks pathogen degradation of an immunity-associated vesicle traffic regulator in
*Arabidopsis*. Proceedings of the National Academy of Sciences.

[R19] Noutoshi Yoshiteru, Kuromori Takashi, Wada Takuji, Hirayama Takashi, Kamiya Asako, Imura Yuko, Yasuda Michiko, Nakashita Hideo, Shirasu Ken, Shinozaki Kazuo (2006). Loss of NECROTIC SPOTTED LESIONS 1 associates with cell death and defense responses in Arabidopsis thaliana. Plant Molecular Biology.

[R20] Oh Hye‐Sook, Collmer Alan (2005). Basal resistance against bacteria in
*Nicotiana benthamiana*
leaves is accompanied by reduced vascular staining and suppressed by multiple
*Pseudomonas syringae*
type III secretion system effector proteins. The Plant Journal.

[R21] Rosado Carlos J., Buckle Ashley M., Law Ruby H. P., Butcher Rebecca E., Kan Wan-Ting, Bird Catherina H., Ung Kheng, Browne Kylie A., Baran Katherine, Bashtannyk-Puhalovich Tanya A., Faux Noel G., Wong Wilson, Porter Corrine J., Pike Robert N., Ellisdon Andrew M., Pearce Mary C., Bottomley Stephen P., Emsley Jonas, Smith A. Ian, Rossjohn Jamie, Hartland Elizabeth L., Voskoboinik Ilia, Trapani Joseph A., Bird Phillip I., Dunstone Michelle A., Whisstock James C. (2007). A Common Fold Mediates Vertebrate Defense and Bacterial Attack. Science.

[R22] Roussin-Léveillée Charles, Lajeunesse Gaële, St-Amand Méliane, Veerapen Varusha Pillay, Silva-Martins Guilherme, Nomura Kinya, Brassard Sandrine, Bolaji Ayooluwa, He Sheng Yang, Moffett Peter (2022). Evolutionarily conserved bacterial effectors hijack abscisic acid signaling to induce an aqueous environment in the apoplast. Cell Host & Microbe.

[R23] Tsutsui Tomokazu, Asada Yutaka, Tamaoki Masanori, Ikeda Akira, Yamaguchi Junji (2008). Arabidopsis CAD1 negatively controls plant immunity mediated by both salicylic acid-dependent and -independent signaling pathways. Plant Science.

[R24] Üstün Suayib, Hafrén Anders, Liu Qinsong, Marshall Richard S., Minina Elena A., Bozhkov Peter V., Vierstra Richard D., Hofius Daniel (2018). Bacteria Exploit Autophagy for Proteasome Degradation and Enhanced Virulence in Plants. The Plant Cell.

[R25] Wang Yan, Pruitt Rory N., Nürnberger Thorsten, Wang Yuanchao (2022). Evasion of plant immunity by microbial pathogens. Nature Reviews Microbiology.

[R26] Weßling Ralf, Epple Petra, Altmann Stefan, He Yijian, Yang Li, Henz Stefan R., McDonald Nathan, Wiley Kristin, Bader Kai Christian, Gläßer Christine, Mukhtar M. Shahid, Haigis Sabine, Ghamsari Lila, Stephens Amber E., Ecker Joseph R., Vidal Marc, Jones Jonathan D.G., Mayer Klaus F.X., Ver Loren van Themaat Emiel, Weigel Detlef, Schulze-Lefert Paul, Dangl Jeffery L., Panstruga Ralph, Braun Pascal (2014). Convergent Targeting of a Common Host Protein-Network by Pathogen Effectors from Three Kingdoms of Life. Cell Host & Microbe.

[R27] Xin Xiu-Fang, Nomura Kinya, Aung Kyaw, Velásquez André C., Yao Jian, Boutrot Freddy, Chang Jeff H., Zipfel Cyril, He Sheng Yang (2016). Bacteria establish an aqueous living space in plants crucial for virulence. Nature.

